# On-farm solid state simultaneous saccharification and fermentation of whole crop forage rice in wrapped round bale for ethanol production

**DOI:** 10.1186/s13068-014-0192-9

**Published:** 2015-01-30

**Authors:** Mitsuo Horita, Hiroko Kitamoto, Tetsuo Kawaide, Yasuhiro Tachibana, Yukiko Shinozaki

**Affiliations:** National Institute for Agro-Environmental Sciences, 3-1-3 Kannondai, Tsukuba, Ibaraki 305-8604 Japan; National Agricultural Research Organization, Bio-oriented Technology Research Advancement Institute, 1-40-2 Nissin, Kitaku, Saitama, Saitama 331-8537 Japan; Current address: National Agricultural Research Organization, Institute of Livestock and Grassland Sciences, 768 Senbonmatsu, Nasushiobara, Tochigi 329-2793 Japan

**Keywords:** Bioethanol, Solid state fermentation, Whole crop forage rice, Round bale, Nutritional value

## Abstract

**Background:**

In an attempt to reduce environmental loading during ethanol production from cellulosic plant biomass, we have previously proposed an on-site solid state fermentation (SSF) method for producing ethanol from whole crops, which at the same time provides cattle feed without producing wastes. During the ensiling of freshly harvested plant biomass with cellulase and glucoamylase, the added yeast and lactic acid bacteria induced simultaneous saccharification and production of ethanol and lactic acid in hermetically sealed containers on-farm. In a previous study, laboratory-scale SSF (using 250 g of fresh rice crop biomass) yielded 16.9 weight % ethanol in dry matter (DM) after 20 days of incubation. In this study, the fermentation volume was scaled up to a normal-sized round bale and the fermentation process (ethanol concentrations of the products) was monitored. The ethanol produced was recovered and the recovery efficiency was evaluated.

**Results:**

SSF tests with forage rice round bales using polyethylene-wrapped whole plant materials (cultivar Leaf Star, average of 125.2 kg dry weight) were monitored in the field without temperature control. They yielded 14.0 weight % ethanol and 2.9 weight % lactic acid in DM after six months of incubation, and the ethanol ratio in the bale remained stable for 14 months after processing. SSF tests with three different rice cultivars were conducted for three years. Ethanol recovery from a fermented whole bale (244 kg fresh matter (FM) containing about 12.4 kg ethanol) by one-step distillation using vacuum distillation equipment yielded 86.3% ethanol collected from distilled solution (107 kg of 10.0 weight % ethanol). In addition, an average of 1.65 kg ethanol in 40.8 kg effluent per bale was recovered. Relative nitrogen content was higher in SSF products than in silage made from the same plant material, indicating that fermentation residue, whose quality is stabilized by the lactic acid produced, can be used as cattle feed.

**Conclusions:**

We have successfully demonstrated an efficient on-site ethanol production system with non-sterilized whole rice crop round bale. However, issues concerning the establishment of the ethanol recovery procedure on-site and evaluation of the fermentation residue as cattle feed have to be addressed.

## Background

The production technique for first generation bioethanol, recognized as a renewable energy source [1], through fermentation of corn and sugarcane is well established. To date, worldwide production volume of bioethanol has been increasing due to policies of many countries which consider bioethanol as one of the substitutes for fossil fuels, which is mostly consumed as liquid fuel for motor vehicles. However, rapid scale-up of bioethanol production has resulted in land use competition with that of animal feed, and has contributed to higher grain prices [1,2]. To avoid such competition, considerable efforts have thus been pursued towards the development of techniques for second generation bioethanol production from lignocellulosic biomass (such as corn stover, wheat straw, rice straw, and wood). However, compared with the already established first generation bioethanol industry, that of second generation bioethanol requires larger facilities, consumes higher amount of energy for transportation of bulky raw materials, and requires pretreatment of indigestible structural polymers, such as cellulose and hemicellulose, to convert them into fermentable sugars, as well as post-treatment of fermentation effluent [2,3].

Instead of a complicated and expensive process, we planned to develop a simple and convenient method for local bioethanol production by applying the silage making process [4]. Silage is the product of natural solid-state lactic acid fermentation of lignocellulosic plant materials with low water content [5]. Silage is a high quality feed for ruminant animals produced with a minimum of harvesting losses of plant materials, regardless of weather conditions. The crops used for silage, which are frequently harvested in their vegetative stage of growth, accumulate nonstructural water-soluble carbohydrates (WSC) within the whole plant. At the early stage of silage making, the lactic acid produced by lactic acid bacteria from WSC kills other kinds of bacteria from plant surfaces and preserves the nutrients of plant materials [5].

On the other hand, for second generation bioethanol production, WSC of plant materials are removed during preservation of materials and in the pretreatment process, such as acid, alkali, or steam- treatment [6]. We hypothesized that WSC of freshly harvested lignocellulosic plant materials would be easier to convert to ethanol compared to plant structural carbohydrates in withered plant materials [4].

To produce silage using crops with comparatively low sugar content, addition of cellulase at ensiling would be useful to release the sugars from structural carbohydrates [7]. Tomoda *et al.* [8] reported that addition of cellulase from *Acremonium cellulolyticus* (0.089 U/g DM avicelase activity) induces lactic acid production (5.7% DM versus 0.5% DM without enzyme) during silage fermentation of alfalfa. Under anaerobic and acidic conditions (pH 3.5 to 6.5) due to lactic acid in silage, the yeast *Saccharomyces cerevisiae* is able to convert sugar to ethanol [5]. Based on these traditional, widely used processes, we devised a way to increase ethanol production during silage fermentation. During ensiling of fresh biomass with biomass-degrading enzymes, exogenous yeast and lactic acid bacteria induce multiple parallel reactions for biomass degradation and ethanol and lactic acid production in solid state fermentation (SSF) in hermetically sealed containers at a ranch [4,9]. Previous laboratory-scale SSF tests (using 250 g of fresh rice whole crop biomass, 0.86 filter paper degradation unit (FPU)/g DM cellulase from *A. cellulolyticus* and 0.32 unit/g DM glucoamylase) yielded 16.9 weight % ethanol in DM at 20 days of incubation [4].

In this study, to validate our designed SSF system, scale-up of the fermentation was carried out by preparing wrapped round bales of non-sterilized whole forage rice plants with enzymes and yeast, and the ethanol production process was monitored. Furthermore, the recovery of ethanol from a fermented round bale was tested.

## Results and discussion

### Production of ethanol and lactic acid during solid state fermentation of forage rice whole crop round bales

**Figure 1:**
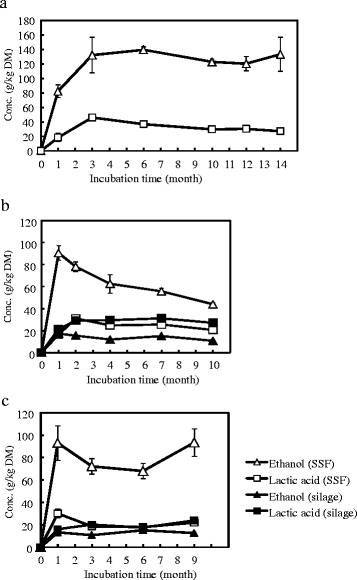
**Solid state fermentation (SSF) using non-sterilized whole forage rice plant round bales.** Ethanol and lactic acid production from forage rice cultivars **(a)** Leaf Star, **(b)** Tachisugata, and **(c)** Tachisuzuka with the addition of 0.74 to 0.77 filter paper degradation unit (FPU)/g dry matter (DM) cellulase from *Acremonium cellulolyticus*, 0.28 to 0.29 U/g DM glucoamylase, freeze-dried lactic acid bacteria (2 × 10^5^ colony forming unit (cfu)/g DM), and freeze-dried yeast (3 × 10^6^ cfu/g DM) mixture (SSF) or freeze-dried lactic acid bacteria only (silage) are shown. Values are expressed as the mean with standard error bars (n = 3 (cv. Leaf Star), n = 2 (cv. Tachisugata), or n = 4 (cv. Tachisuzuka)).

Ethanol and lactic acid content in unsterilized forage rice round bales (cv. Leaf Star, average DM weight of three bales was 125.2 kg) during SSF are shown in Figure 1a. After one month of incubation, 82.3 g/kg DM ethanol had accumulated in the round bales. The ethanol content continued to increase until the third month of incubation, with the maximum average concentration of 139.6 g/kg DM reached after six months of incubation. The ethanol concentration was comparatively stable until the end of the test (14 months of incubation). The lactic acid concentration reached a maximum of 46.0 g/kg DM after three months of incubation, and was relatively stable (>27.1 g/kg DM) until the end of the test. In addition, accumulation of effluent from the bale was observed throughout the incubation period, and was accompanied by a marked decrease in the DM of the bale, losing 34.4% weight in DM at three months (data not shown), and 39.9% at 14 months of incubation (Table 1), respectively.

**Table 1:** **Weight loss of solid state fermented (SSF) and silage round bales**

**Cultivar**	**Start**	**End**	**Weight loss**	
	**(kg dry matter (DM))**	**(kg DM)**	**(kg DM)**	**(%)**
Leaf Star (SSF)	125.2	75.2	50.0	39.9
Tachisugata (SSF)	146.9	104.3	42.6	29.0
Tachisuzuka (SSF)	151.4	107.8	43.6	28.8
Tachisugata (silage)	140.3	118.8	21.5	15.3
Tachisuzuka (silage)	150.9	127.2	23.7	15.7

The reproducibility of these results was confirmed by conducting SSF tests with other forage rice cultivars, using one each year. In the second (October 2010 to August 2011, cv. Tachisugata, Figure 1b) and third (November 2011 to August 2012, cv. Tachisuzuka, Figure 1c) SSF tests, the maximum ethanol concentrations were 90.7 g/kg and 92.9 g/kg DM after one month of incubation, respectively. Thereafter, their ethanol concentrations decreased. On the other hand, average lactic acid production in the bale of Tachisugata and Tachisuzuka was 16.5 g/kg and 30.1 g/kg DM, respectively, after one month of incubation. Furthermore, over 16.5 g/kg lactic acid in DM had been kept in the bales of both cultivars during the entire incubation period.

In order to compare the production rates of ethanol and lactic acid in our SSF system with those of normal silage, round bale without any added enzymes and yeast was also prepared, and monitored in the second (cv. Tachisugata, Figure 1b) and third (cv. Tachisuzuka, Figure 1c) tests. The maximum ethanol concentrations produced in silage bales were 17.3 g/kg and 15.4 g/kg in DM, respectively, after between one and three months of incubation. The ethanol and lactic acid contents of silage were over 11.0 g/kg and 16.0 g/kg in DM, respectively, throughout the incubation period. This could be due to the fact that wild yeasts usually inhabit forage crops and frequently produce small amounts of ethanol in normal silage bales [5]. Similarly, wild yeasts present in the forage rice plants used in this study could be expected to produce ethanol.

In our SSF study using round bales, a steady amount of ethanol (>80 g/kg DM) was observed to have accumulated at a short incubation period (one month) in all tested bales regardless of cultivars used. However, with longer incubations, the ethanol content of the bales showed differences among the forage rice cultivars used, with the highest observed in the Leaf Star bale, and the lowest in the Tachisugata bale. As mentioned previously, plants frequently accumulate WSC in the leaves and stems at the vegetative stage, and these WSC are converted to lactic acid during silage fermentation [5]. These results suggested that during SSF of round bale, inoculated enzyme degraded the carbohydrate polymers (such as cellulose, hemicellulose, and starch) in the plant structure, causing the release of additional fermentable sugars in the bale, thus enhancing the production of lactic acid and ethanol.

Forage rice cultivars developed for whole crop silage in Japan were selected based on their ability to accumulate WSC. Tachisugata is one of the selected rice cultivars because of its high whole crop productivity and high brown rice yield (40% DM of the whole crop) [10]. However, the excretion of indigestible unhulled rice is attributed to the low digestibility of rice whole crop by cattle [11].

In contrast, Leaf Star and Tachisuzuka were recently developed forage rice cultivars that were selected for their high straw biomass yield, and their low brown rice yield of 26% and 13% DM, respectively [12,13]. In addition, compared to other cultivars, Leaf Star has higher WSC in the stem and leaf sheath and lower lignin content [12], which is the substrate of biomass-degrading enzymes. During SSF, differences in the above characteristics among rice cultivars could have influenced the amount of sugar released, and consequently, the ethanol production by yeast.

### Distribution of ethanol, lactic acid, and moisture in the round bale

At the end of fermentation test, the bales were opened and samples were taken and analyzed for their distribution of ethanol, lactic acid, and moisture, as well as pH (Table 2).

**Table 2:** **Ethanol and lactic acid concentration, moisture, and pH in solid state fermented (SSF) round bale of whole forage rice plant**

**Cultivar**	**Bale number**	**Sampling site**	**Ethanol (g/kg DM)** ^**a**^	**Lactic acid (g/kg DM)** ^**a**^	**Moisture (%)** ^**a**^	**pH**
Leaf Star	1	Upper	128.7^x^	24.8^x^	68.6^x^	3.96
		Middle	132.6^x^	29.9^x^	67.2^x^	3.93
		Lower	117.1^x^	26.7^x^	63.7^x^	3.99
	2	Upper	155.3^x^	29.7^x^	68.0^x^	4.03
		Middle	178.7^x^	31.4^x^	70.4^x^	4.00
		Lower	169.3^x^	31.0^x^	69.7^x^	3.99
Tachisugata	1	Upper	42.4^y^	19.5^y^	59.0^xy^	4.12
		Middle	45.4^y^	21.8^xy^	57.5^y^	4.10
		Lower	178.5^x^	26.9^x^	64.2^x^	3.90
Tachisuzuka	1	Upper	60.5^y^	19.5^y^	58.1^y^	4.07
		Middle	72.5^y^	19.4^y^	62.0^y^	4.08
		Lower	133.8^x^	48.6^x^	72.0^x^	3.91
	2	Upper	72.5^y^	23.1^y^	62.3^xy^	4.11
		Middle	90.9^y^	23.5^y^	61.9^y^	4.12
		Lower	143.4^x^	38.8^x^	68.3^x^	3.95

Whole rice plant round bales (about 0.8 m height and 1 m diameter) after each SSF test (9 to 14 months’ incubation) were opened and three samples from each of the three distinct height sampling sites (upper (>60 cm), middle (30 to 60 cm), and lower (<30 cm)) in the round bale were collected individually, and ethanol and lactic acid concentration, moisture, and pH were analyzed.

^a^Different alphabetical letters following the number indicate significant differences (*P* <0.05, Scheffe’s test) among sampling sites within the same round bale.

In the SSF bales incubated for 14 months used in the first test (cv. Leaf Star), high concentrations of ethanol (117.1 to 132.6 g/kg DM in bale number one and 155.3 to 178.7 g/kg DM in bale number two) were detected in the evaluated sections (upper, middle, or lower section). On the contrary, in the SSF bales incubated for 10 months used in the second test (cv. Tachisugata), a significantly higher concentration of ethanol (178.5 g/kg DM) was detected at the lower section than that at the upper (42.4 g/kg) and middle (45.4 g/kg) sections. Similarly, in the SSF bales incubated for nine months used in the third test (cv. Tachisuzuka), ethanol concentration at the lower section (133.8 to 143.4 g/kg DM) was much higher than that at the upper (60.5 to 72.5 g/kg) and middle (72.5 to 90.9 g/kg) sections.

These results showed that among the sections of Tachisugata and Tachisuzuka round bales (Table 2), the lower section showed significantly higher ethanol and lactic acid concentrations and water contents than those at the upper and middle sections. In general, the round bale was wrapped with a multilayer gas and water impermeable film, and the natural convection of free water inside it was induced under repeated daily changes in temperature [5]. We speculated that cooled water droplets could have washed away the ethanol and lactic acid from the fermented products during repeated convection, and subsequently these materials became concentrated and stored at the bottom of the Tachisugata and Tachisuzuka round bales.

As shown in Figure 1, ethanol concentrations of Tachisugata and Tachisuzuka round bales decreased after one month of incubation. We speculated that the above phenomenon (natural convection of free water) could have affected the distribution of the ethanol inside the round bale and caused the decrease in the ethanol concentration at the sampling site (middle section), in addition to the cessation of ethanol fermentation and the natural release (evaporation or leakage as effluent) of accumulated ethanol to outside.

In comparison, the physical water holding capacity of the bale made from Leaf Star was comparatively higher than other bales, thus enabling it to retain impartially the produced ethanol, lactic acid, and moisture inside.

### Transition of round bale temperature during solid state fermentation

**Figure 2:**
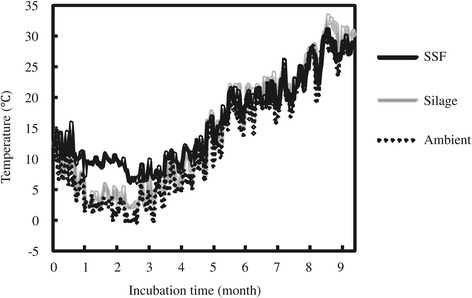
**Variation of temperatures inside and outside of solid state fermented (SSF) and silage round bales.** Daily average temperatures inside (30 cm depth) and outside (ambient) of the SSF and silage round bales (cv. Tachisuzuka) throughout the fermentation period (November 2011 to August 2012) are shown.

Temperatures inside the SSF round bales (cv. Tachisuzuka), a normal silage bale, and outside air throughout the fermentation period (November 2011 to August 2012) are shown in Figure 2. The ambient temperature decreased to about 2°C after one month of incubation. Simultaneously, the temperature inside the silage round bale also gradually fell to about 2°C. On the contrary, the average temperature inside the three SSF round bales remained relatively higher than those of silage and outside bales until the third month of incubation. They were stable at 10 to 14°C until the second month after bale preparation, then gradually dropped to that of the atmosphere until it reached the ambient temperature (6°C) at the third month. Thereafter, the temperature of all bales was almost the same as that of the atmosphere. After five to six months of incubation (April to May), all test bales increased in temperature to over 20°C. The temperatures of the second (cv. Tachisugata) fermentation test round bales were also measured, and were found to show diverse patterns almost similar to those of the third test (data not shown).

The temperature of SSF round bales remained high (>10°C) until the second month of incubation because the additive yeast and lactic acid bacteria could have metabolized the saccharides (substrates) resulting from the activity of the biomass-degrading enzymes. From the second month, the temperature inside and outside the bales had become the same. As shown in Figure 1c, ethanol concentration in the bale did not increase after three months. This implied that the metabolic activity mentioned above had gradually ceased. In winter season, the sudden decline of the temperature outside the bale likely reduced the activities of the biomass-degrading enzymes, as well as the ethanol fermentation activity of the yeast in the bale. It indicated the possibility that keeping the bale warm (>15°C) at the early stage of the SSF process would further facilitate ethanol production in the bale.

### Weight loss during solid state fermentation

Weight loss of SSF round bales and silage bales before and after fermentation are shown in Table 1. All SSF round bales greatly decreased in DM weights (42.6 to 50 kg, 28.8 to 39.9%). Silage round bales (cv. Tachisugata and cv. Tachisuzuka) also decreased in DM weights (21.5 to 23.7 kg, 15.3 to 15.7%). These results indicated that the fermentation profile of SSF is comparatively similar to that of silage fermentation.

It is well known that a significant amount of DM weight (between 12 and 20%) is lost by respiration and fermentation, and that effluent (<10%) has been found during ensilage [5]. In this study, an average of more than 15% DM weight loss was observed in normal silage round bales.

In the ensiled wet crop material with very high WSC, the lactic acid bacteria in the silage were extremely activated, resulting in the enhancement of lactic acid fermentation [5]. Moreover, the addition of cellulase apparently increased the WSC content of herbage, and consequently caused an increase in the lactic acid accumulation in silage [14]. Similarly, in the SSF round bale, large amounts of solid carbohydrates in plant biomass are expected to have been converted to water soluble constituents (such as saccharides, ethanol, and lactic acid) by the activity of inoculated biomass-degrading enzyme, yeast, and lactic acid bacteria.

In this study, compared with normal silage bales, further DM weight losses in the SSF round bales (13.7% (29.0 − 15.3) in cv. Tachisugata bales and 13.1% (28.8 − 15.7) in cv. Tachisuzuka bales) were observed. In addition, maximum average ethanol concentrations in cv. Tachisugata and cv. Tachisuzuka SSF round bales greatly increased (7.4 (9.1 − 1.7) and 7.8 (9.3 − 1.5) weight % in DM, respectively) (Figure 1). These results suggested that about 13 to 14% DM weight losses in cv. Tachisugata and cv. Tachisuzuka SSF round bales was caused by additive enzymes and microbial activities.

Effluent is produced during fermentation of silage made from plant materials with high moisture content (>60%) [5]. Due to natural convection, the moisture in the bale that accumulated at the bottom of the wrapped round bale drained naturally as effluent. Silage effluent contains high amount of nutrients, and has a potential for animal feed use [5]. The SSF effluent obtained in this study was also abundant in saccharides (0.2 kg hexose (glucose, fructose, and sucrose), 0.14 kg xylose) and lactic acid (0.53 kg) per round bale.

### Composition of effluent obtained from the solid state fermented round bale

**Figure 3:**
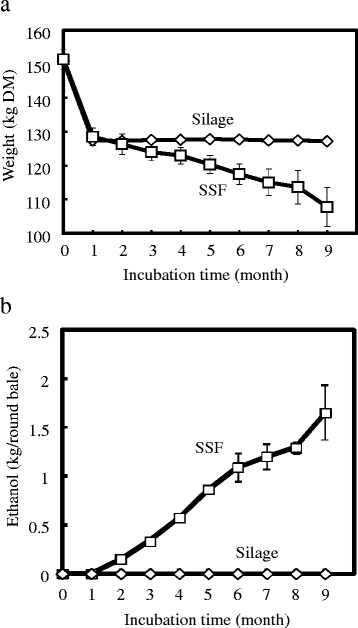
**Loss of solid state fermented (SSF) and silage round bale weight and amount of ethanol recovered with drain. (a)** The round bale weight (DM) and **(b)** the amount of recovered ethanol during the SSF test were measured every month. Values are expressed as the mean with standard error bars (n = 4). DM, dry matter.

Because of the apparent weight reduction observed during the first SSF test (Table 1), the whole round bale in the second and third test was further enclosed in a water-impermeable plastic bag, and the effluent that drained out was collected at the bottom of the bale. The DM weight loss of Tachisuzuka (SSF and normal silage) round bale and the ethanol content of the effluent are shown in Figure 3. After one month of incubation, their average DM weight greatly declined (−23.9 kg). From the second to the ninth month, the DM weight gradually decreased. Similarly, the average DM weight of silage round bales decreased (−23.5 kg) after one month, but their weight remained stable thereafter.

The maximum average DM weight loss of the SSF round bale in the first month was closely correlated with the high temperature in the SSF round bale (Figure 2), which might be caused by metabolic heat of additive microorganisms, and the highest rate of ethanol and lactic acid production (Figure 1). It indicated that a major part of lost DM weight of round bales had been converted to water soluble constituents (such as ethanol, lactic acid, and saccharides).

Effluent was successively recovered from the second to the ninth month. The recovered effluent always contained 3.5 to 4.3 weight % of ethanol. The total amount of recovered effluent was 40.8 kg/round bale, yielding an average of 1.65 kg ethanol (Figure 3).

The decrease of average DM weight of SSF round bales starting from the second month would suggest continuous degradation of solid materials by biomass-degrading enzymes, as evidenced by the comparatively higher temperature (>10°C) inside the SSF round bale and the draining of excess water from the round bale as effluent. Likewise, continuous DM weight reduction was also observed at the second SSF test with Tachisugata round bale (data not shown). The presence of various nutritive components (such as saccharides and lactic acid) in the SSF effluent makes it not only a potential source of ethanol, but also a potential feed for farm animals.

### Recovery of ethanol from the fermented products using vacuum distiller

Results of practical ethanol recovery tests from the SSF whole round bale (cv. Leaf Star) using a vacuum distillation equipment are shown in Table 3. The first recovery test was carried out in a six-hour distillation period using 232 kg fresh matter (FM) of fermented products that contained 5.3 weight % of ethanol (total ethanol content in a round bale was calculated as 12.3 kg). A total of 47.6 kg of ethanol solution (10.9 weight %) was recovered from the bale, and yielded 5.2 kg ethanol. Therefore, this amounted to a recovery equivalent to 42.3% of theoretical ethanol in the round bale. However, after the distillation, a total of 4.0 kg ethanol still remained in the distillation residue. Considering the result of the first test, the second test was carried out with a longer (12 hours) distillation period using 244 kg FM of fermented products that contained 5.1% ethanol (total ethanol content was calculated as 12.4 kg). As a result, 107.2 kg of ethanol solution (10.0 weight %) was recovered, which yielded 10.7 kg of ethanol (86.3% of theoretical ethanol in the round bale). Consequently, a total of 15.9 kg ethanol was recovered from 476 kg FM of fermented products (that contained 24.7 kg ethanol), which was equivalent to an average ethanol recovery ratio of 64.6%.

**Table 3:** **Ethanol recovery from solid state fermented (SSF) round bales using a vacuum distiller**

	**SSF round bale**		**Ethanol recovery**		
	**Weight (kg fresh matter)**	**Ethanol (kg)**	**Ethanol (kg)**	**Recovery rate (%)**	**Distillation time (hours)**
First	232	12.3	5.2	42.3	6
Second	244	12.4	10.7	86.3	12
Total	476	24.7	15.9	64.4	18

Ethanol recovery test from SSF round bales of whole forage rice plant (cv. Leaf Star) using a pilot-scale vacuum distiller (Tokai Resource Co.) was repeated two times with distinct distillation times (6 and 12 hours), and the amounts of recovered ethanol were compared.

### Analysis of nutritional value of the solid state fermented product

The nutritional value of SSF product, the plant material used (freshly harvested rice whole plants), and silage made from the same material are listed in Table 4. The data showed that lactic acid content and pH of the SSF product are similar to those of silage. Keeping the pH low (around 4.0) by lactic acid in silage is an important factor for long-term preservation [5]. Similarly, SSF residue could also be expected to avoid spoilage for comparable periods of time.

**Table 4:** **Composition of fresh, silage, and solid state fermented (SSF) materials of forage rice plants (cv. Tachisuzuka)**

**Composition (%)** ^**a**^	**Fresh material**	**Silage**	**SSF product (residue)**
Moisture	56.2	63.9	64.7
Weight loss^b^	-	15.7	28.8
Cellulose^c,d^	22.0	22.1 (18.6)^g^	22.0 (15.7)
Hemicellulose^d, e^	15.2	12.1 (10.2)	13.8 (9.8)
Lignin^d^	2.9	3.4 (2.9)	4.0 (2.8)
Starch^d^	18.9	20.2 (17.0)	5.3 (3.8)
Hexose^f^	5.4	0.4 (0.3)	7.6 (5.4)
Xylose	0.0	0.5 (0.4)	1.7 (1.2)
Lactic acid	0.0	2.4 (2.0)	3.4 (2.4)
Total carbon	34.34	37.29 (31.44)	36.77 (26.20)
Total nitrogen	0.47	0.73 (0.62)	1.08 (0.77)
Carbon:nitrogen	72.9	51.4	34.1
pH	5.68	4.17	4.00

^a^Percent by dry matter.

^b^Weight loss by dry matter during fermentation process (Table 1).

^c^Acid detergent fiber (ADF) - lignin.

^d^Composition analysis was carried out by Japan Food Research Laboratories.

^e^Neutral detergent fiber (NDF) - ADF.

^f^Glucose + fructose + sucrose.

^g^Numbers in parentheses indicate the composition (%) × (100 - weight loss (%))/100.

The ratios of cellulose and hemicellulose in the total dry matter of SSF product and silage were not much different from those of fresh whole plant. When the 28.8% dry weight loss during SSF was taken into account (Table 1), the cellulose and hemicellulose contents of the fresh whole plant decreased by 6.3% and 5.4%, respectively. On the contrary, the starch content in the SSF product was much lower than that in fresh plant, while the saccharides (hexose + xylose) and lactic acid contents in the SSF product were comparatively higher.

These results suggested that in SSF prepared with cellulase and glucoamylase, a major part of the saccharides (glucose, fructose, and sucrose) were supplied from the degraded starch and cellulose in plants, which were then converted to ethanol or lactic acid by the activities of added yeast and lactic acid bacteria. However, the degree of degradation of cellulose and hemicellulose were observed to be relatively low. These results coincided with those of the laboratory-scaled fermentation [4]. However, the production of SSF prepared without cellulase showed a similar fermentation profile with that of silage (data not shown). The commercially available cellulase from *A. cellulolyticus* used in this study contains significantly high pectinase activity and is effective for the degradation of ensiled rice straw [9]. It suggests that the different activities of the cellulase have an important role in opening access points to the plant structure that covers the leaves and stems where WSC are contained, and thus facilitating starch degradation by glucoamylase.

Regarding the relative nitrogen content (% dry weight) in the SSF product, it was found to have doubled (1.08% versus 0.47%) over that of the fresh whole plant, and the content was higher than that in silage (0.73 weight %) (Table 4). Similarly, in the laboratory-scaled SSF test, we also reported that a large decrease in the amylase-degradable fraction had resulted in a greater amount of crude protein in fermentation residue compared with silage [4]. It was assumed that exogenous yeast and lactic acid bacteria supplied for the SSF test would multiply inside the round bale, and the cells themselves would become the nitrogen source. Since crude protein is an essential diet component for cattle, the nutritional composition of fermentation residue indicates that it is suitable for animal feed.

## Conclusions

Generally, the problem areas in ethanol production from cellulosic materials are as follows: 1) the transportation of bulky materials to large factories, 2) loss of nutrients (such as enzymatically degradable cellulose, starch, and saccharides) from biomass during preservation which are essential for bioethanol production, 3) pretreatment (acid, alkali, or steam) before fermentation which consumes a large amount of energy, and 4) the recycling of large amounts of effluents after ethanol distillation [15]. In our SSF system, harvested materials are immediately packed into a round bale in the field, which is similar to a conventional silo used for silage fermentation and does not require special facilities. Immediately after packing harvested materials into the round bale, free sugars and enzymatically digestible carbon in the biomass are converted to ethanol and lactic acid during preservation. Although the said SSF system requires a relatively long time for degradation and fermentation, no energy needs to be supplied to the system, and farmers are familiar with preserving silage on their farms.

In this study, we assessed the feasibility of SSF using forage rice plant round bales without temperature control. Because the preparation process was almost the same as for silage fermentation, it had a favorable start. The results showed that a steady amount of ethanol (90.9 to 139.6 g/kg DM) was produced and accumulated in the round bale within one to six months of incubation. Depending on the rice cultivar used, the maximum ethanol concentration that accumulated in the round bale was stably maintained for more than one year. Furthermore, similar to the silage round bale, more than 16.5 g/kg DM of lactic acid was produced and had accumulated together with ethanol in the fermented round bale.

In this SSF process, the recovery condition for ethanol is different from that in a normal liquid ethanol fermentation system since ethanol evaporation using hot steam, which is common for the separation of ethanol from the fermentation liquid, was not available for SSF material with a low water content. We also examined the ethanol recovery from the SSF round bale using vacuum distillation equipment that has been designed for ethanol recovery from SSF food residue. With this recovery system, a maximum of 86.3% (10.7 kg) of ethanol could be recovered from a 244 kg (FM) fermented round bale. These results indicated the large potential of our SSF system for the practical production and recovery of ethanol.

Moreover, a steady amount of ethanol continuously drained out in the effluent from the SSF round bale during the test. The amount of ethanol contained in the effluent was 16% of produced ethanol in our SSF system. Thus, the recovery of ethanol from the effluent would highly increase the total amount of recovered ethanol from the fermented round bale. In addition, since the major nutritional components essential for raising cattle (cellulose, hemicellulose, protein, lactic acid, and so forth) remained in the SSF residue, it could be used as feed for cattle.

Because most of the ethanol contained in the SSF round bale could directly be recovered as ethanol solution without insoluble particles, it could be easily dehydrated and concentrated for use as automotive fuel and a biochemical resource. Thus, our new findings suggest that the SSF of cellulosic plant biomass could provide a feasible solution to the problems of the normal bioethanol production system.

Further studies such as improvement of ethanol production yield (at present, a maximum of 14.0 weight % in DM) and the ethanol recovery ratio, evaluation of fermentation residue for cattle feed, and life cycle assessment (total eco-balance) should also be carried out in order to successfully establish a practical solid-state ethanol fermentation system in rural areas.

## Methods

### Preparation of the forage rice plants round bale

Three forage rice cultivars (cvs. Leaf Star, Tachisugata, and Tachisuzuka) were selected and used for the test based on their ability to accumulate WSC [10,12,13]. These were cultivated at a paddy field in the National Agricultural Research Organization, Bio-oriented Technology Research Advancement Institution (Konosu, Saitama, Japan) from May to October or November of the test year. These forage rice plants were used for SSF tests in preparing round bales. For the purpose of convenient silage production from forage rice plant, facilities and equipment for round bale production had been systematically introduced in Japan, and making the use of round bales popular. The SSF tests were carried out three times across four years (November 2009 to August 2012), using one cultivar for each test. In the first test (16 November 2009 to 8 February 2011), whole forage rice plants (cv. Leaf Star) at the vegetative stage were harvested using a forage harvester (SMR1000, Takakita Co., Nabari, Japan), and were chopped (3 to 5 cm length, 273 to 283 kg, 48 to 50 dry weight %) and mixed with biomass-degrading enzymes (0.74 to 0.77 FPU/g DM cellulase from *A. cellulolyticus* (Meiji Seika Pharma Co., Tokyo, Japan) and 0.28 to 0.29 U/g DM glucoamylase from *Aspergillus niger* (GODO-ANGH, Godo Shusei Co., Tokyo, Japan)), freeze-dried lactic acid bacteria (2 × 10^5^ cfu/g DM, Chikuso No.1 for silage additive, Snowseed Co., Sapporo, Japan), and freeze-dried *S. cerevisiae* (3 × 10^6^ cfu/g DM, Saf-instant, S. I. Lesaffre, Marcq, France) dissolved in 40 kg of distilled water. The amounts of these additives were determined based on our previous report [4].

Filter paper degradation activity of saccharifying cellulase (FPU assay) and the activities of glucoamylase were measured as described previously [9]. Thereafter, the forage rice round bale (ca. 0.8 m in height, 1 m in diameter) was prepared by a round baler (Takakita Co.) and an automatic bale wrapper (Takakita Co.) with multi-winding white plastic film for silage (Silo Fix, Hokuetsu Kasei Co., Mitsuke, Japan). Preparation of round bale for the second (cv. Tachisugata, 14 October 2010 to 18 August 2011) and third (cv. Tachisuzuka, 17 November 2011 to 24 August 2012) fermentation tests were carried out similarly as in the first test. As a control for the second and third SSF tests, silage round bales were prepared from the same plant material added only with freeze-dried lactic acid bacteria.

### Solid state fermentation in the field and sampling

After measuring the weight of round bales, they were left on the field and the solid-state ethanol fermentation test was conducted without controlling the temperature. The temperatures inside (30 cm depth) and outside (ambient) of the multiple round bales were recorded every day using thermo-recorders (TR-72Ui, T&D Co., Matsumoto, Japan) and thermo-sensors (TR-0506, T&D Co.).

The weight of the round bale was measured at one to three month intervals. Simultaneously, a few points of the middle section (30 to 60 cm height) of the bale were gouged out to 25 cm depth by a feed sampler (Fujiwara Scientific Co., Tokyo, Japan), and each collected sample (ca. 300 g) was preserved at 4°C until use in a gas and water impermeable plastic bag lined with aluminum film (AL-30 L, Seisan Nippon Co., Tokyo, Japan). Equal amounts of each sample in the same bale was mixed and used for analysis.

To collect the SSF effluent, each round bale was further enclosed in a bag of water-impermeable polyethylene plastic film (FTX3500, Showa Paxxs Co., Tokyo, Japan). The effluent collected at the bottom of the bag was recovered monthly; its weight was measured and its major components (ethanol, lactic acid, acetic acid, and saccharides) were analyzed.

### Analysis of the fermented products

After sampling of the fermented products, a 5 g portion of each sample was mixed with sterilized distilled water four to 10 times its weight and shaken for 30 minutes at 170 rpm at room temperature. The extracted solution was analyzed for ethanol, sugar (glucose, fructose, and sucrose), D- and L-lactic acid, and acetic acid content using an enzymatic method (F-kit, R-Biopharm AG, Darmstadt, Germany) according to the manufacturer’s instructions. Similarly, D-xylose was quantified using the D-xylose assay kit (Megazyme International Ireland, Wicklow, Ireland). The SSF effluent samples were also analyzed. All chemicals used were analytical grade and obtained from Sigma-Aldrich (St Louis, Missouri, United States) or Wako Pure Chemical Industries (Osaka, Japan). To measure the dry weight % of each sample, 10 g of each material was dried overnight at 70°C in a forced-air drying oven (O-190FDS, Sunaka Rika Kogyo, Tokyo, Japan) [4,9].

### Ethanol recovery from solid state fermented whole rice plants using a vacuum distiller

Ethanol recovery tests were performed using a pilot-scale vacuum distiller, which was designed for ethanol recovery from SSF food residue (Tokai Resource Co., Nagoya, Japan). After removing the plastic film wrapped around the SSF whole rice plants round bale incubated for 14 months, the fermented material (232 to 244 kg FM, 5.1 to 5.3 weight % ethanol on a wet basis) was stirred in a reactor (800 L volume) under evaporation (−0.1 MPa), and was incubated in a microwave and hot steam heating furnace until 60°C was reached. Then the vapor produced in an adjunct distillation tower was collected at 4°C and maintained using a chiller. The test was repeated two times. Distillation times (including the amount of time it took to reach 60°C) for the first and second tests were estimated to be six hours and 12 hours, respectively.

After the test, the amount of the recovered solution and its ethanol concentration, total and dry weight of the fermentation residue, amount of residual ethanol in the residue, and ethanol recovery ratio were measured and calculated.

### Analysis of the nutritional value of the solid state fermented residue

Measurements of the cellulose, hemicellulose, lignin, and starch content of the SSF round bale, silage, and fresh whole forage rice plant materials were carried out by Japan Food Research Laboratories (Tokyo, Japan) (test results number: 12088086002–01). Before the analysis, the samples were dried in a forced-air drying oven (O-190FDS, Sunaka Rika Kogyo) at 70°C overnight, powdered using a laboratory-scale mill (A11BS1, IKA, Staufen, Germany), and passed through a 2-mm mesh. The analyses were done based on the method of Van Soest and McQueen [16]. Cellulose and hemicellulose content were calculated as acid detergent fiber (ADF) - lignin, and neutral detergent fiber (NDF) - ADF, respectively. Total carbon, total nitrogen, and carbon-to-nitrogen ratio were measured and calculated using an automatic highly sensitive nitrogen and carbon analyzer (Sumigraph NC-22 F, Sumika Chemical Analysis Service, Ltd., Osaka, Japan) according to the manufacturer’s instruction.

## Abbreviations

ADF: Acid detergent fiber
cfu: Colony forming unit
DM: Dry matter
FM: Fresh matter
FPU: Filter paper degradation unit
NDF: Neutral detergent fiber
SSF: Solid state fermentation
WSC: Water-soluble carbohydrates
